# Thermal conductivity and thermal diffusivity of fullerene-based nanofluids

**DOI:** 10.1038/s41598-022-14204-y

**Published:** 2022-06-10

**Authors:** Brian Reding, Mohamed Khayet

**Affiliations:** Department of Structure of Matter, Thermal Physics and Electronics, Faculty of Physics, University Complutense of Madrid, Avda. Complutense s/n, 28040 Madrid, Spain

**Keywords:** Engineering, Materials science, Nanoscience and technology, Physics

## Abstract

Owing to their outstanding characteristics, carbon based nanofluids (CbNFs) have been applied to various advanced heat transfer and cooling technologies. It was claimed that these CbNFs can considerably improve the properties of the base working fluids. Among all the thermal characteristics, the thermal conductivity (*λ*) is regarded as the primary parameter to be considered for the application of nanofluids (NFs). In the present research study we measured for the first time both *λ* and thermal diffusivity (*a*_*T*_) of very stable fullerene (C_60_)-based NFs in liquid phase (1,2,3,4-tetrahydronaphthalene and 1,2-dicholorobenzene) by the transient multi-current hot wire technique at atmospheric pressure in a wide range of temperature (254–323 K). Similar to the base liquids (BLs), we observed a slight decrease in *λ* with an increase in temperature. Additionally, compared to the BLs *λ* was reduced upon the addition of C_60_. The results were compared with the predicted ones using different theoretical models. Not much variation in *a*_*T*_ was observed between the C_60_ NFs and the corresponding BLs due partly to the small variation of *λ* with the addition of C_60_.

## Introduction

Carbon based family of nanomaterials (CbNFs) comprising of single- and multi-layer graphene, single- and multi-walled carbon nanotubes, graphite, graphene nanoplatelets, graphene quantum dots, graphene oxide, and so on have recently received major attention for the preparation of NFs (i.e. dispersion of CbNFs in base liquids, BLs) because of their various excellent and unique characteristics along with their superior thermal attributes as compared to conventional liquids^1–5^. The way the CbNFs bond to and through the molecules of the BLs govern the properties like rheology, thermal and electrical conductivity or light absorption/emission. These NFs have exhibited great potential in industrial applications such as solar thermal storage, heat pipes, and energy storage among other advanced heat transfer and cooling technologies; which is attributable to their greater thermal conductivity (*λ*) and convective heat transfer coefficients compared to the corresponding BLs^2,5^. In fact, *λ* improvement is the first expected advantage of using a nanofluid (NF) when applied as a thermal working fluid. When evaluated against metals or metal oxides (Au, Ag, Cu, Fe, CuO, Al_2_O_3_, ZnO, etc.), *λ* in carbon nanostructures is greater due to their high intrinsic *λ*, low density, strong C–C covalent bonds, and phonon scattering^6^. For instance, *λ* of carbon materials has a wide range that varies from 0.2 W/m K for diamond-like carbons to 6000 W/m K for single-walled carbon nano-tube (SWNT)^7^; which is superior to that of graphene (5300 W/m K for)^8^, double-walled carbon nano-tube (DWNT, 3986 W/m K) and multi-walled carbon nano-tube (MWNT, 3000 W/m K)^5,7,9^. Therefore, carbon materials can be applied either as heat insulators (e.g. diamond-like carbons) or heat superconductors (e.g. graphene).

It is worth noting that various experimental and theoretical studies have reported *λ* enhancement of NFs and the related affecting factors. The results indicated that *λ* of NFs are normally functions not only of the particle’s thermal conductivity, its concentration in a NF, size and shape, but also the environmental parameters such as the base fluid’s, pH value, surfactant, dispersing agent, and the standing time^10^. Various investigations have shown that higher *λ* enhancement of NFs could be obtained when lower *λ* base fluids were considered^2^. In addition, it has been reported that *λ* of NFs is atypically enhanced with a very low volume fraction of nano-additives^11–14^. For instance, in a first study by Choi et al.^12^, a 160% *λ* enhancement for 1.0 vol% multiwall carbon nano-tube (MWCNT) dispersed in synthetic poly (alpha-olefin) (PAO) oil was reported. Much lower *λ* enhancement (i.e. an order of magnitude smaller, as low as 7%) of carbon nano-tubes-based NFs were found in various other studies including functionalized MWCNT and different based fluids (water, oil, decene, ethylene glycol, glycerol, Refrigerant R113, etc.)^14–23^. In some studies, a decrease in time of *λ* was observed, especially by the first 10 days from the preparation of the NF, but the reduction rate also decreased with time^9^.

Contrastingly, some have reported that NFs containing graphene nanomaterials frequently exhibited greater *λ* compared with those including other nano-particles^5^. Yu et al.^24^ stated an increase of *λ* up to 86% for graphene and graphene oxide (GO) based ethylene glycol NFs (i.e. *λ* of the GO NF and graphene NF was 4.9 W/m K and 6.8 W/m K, respectively). Lower *λ* enhancements (below 27%) were claimed in other graphene nanosheets, graphite nano-particles and graphene quantum dots (GQD) based NFs prepared with water or ionic liquids^3,25–27^. It was indicated that, other than the presence of structural defects, inadequate stability, and restacking of graphene oxide, graphene nano-sheets are prone to be coiled, folded, and corrugated at the surface and edges affecting the heat transfer mechanisms and the subsequent measured *λ*.

Among the above cited CbNFs, much less attention has been given to nano-diamond particles and fullerene based NFs^19,28–30^. This may be attributed to the lower *λ* of these nanoparticles. For nano-diamond-water based NFs, the stated *λ* enhancements were up to 22.8%^29,30^. For fullerene C_60_ NFs, it was claimed 6% enhancement of *λ* for oil base liquid; but 3% reduction of *λ* for water base liquid, and a decrease of *λ* with increasing C_60_ volume fraction^19,28^.

In general, there exists a considerable scattering in the measured *λ* by different research groups for the same kind of NFs and even for the same volume fraction of nano-additive having the same size. Although an increase in the volume fraction of the nano-additive seems to always have a positive effect on *λ* of NFs, the enhancement rate is rather dissimilar in different research studies. Such disagreements are attributed to the different sample preparations and stabilization methods. The use of suitable surfactant(s) or dispersing agent(s) to prepare stable NFs, since the nano-additives can form aggregates because of strong van der Waals interactions, not only increases the viscosity but also reduces *λ* of NFs^31–34^. Moreover, even if *λ* of the nano-particle can directly affect *λ* of NFs (i.e. for a similar nano-particle content in a given NF, higher *λ* of the particle generally produces a greater enhancement in *λ* of the NF), this is not conclusive since *λ* of NFs is also affected by other aspects such as aggregation, Brownian motion, interfacial nano-layer, surface charge state, and thermal resistance of nanoparticles, among others^10^.

It must also be mentioned that some research studies have revealed an anomalous *λ* increment of some NFs with an increase of the nano-additive volume fraction within the base fluid^12,35^. This is still a controversial issue^10,36^. The measured *λ* was found to be significantly higher than the theoretical value predicted by the classical Maxwell model^37^ as adapted by Hamilton and Crosser^38^. This was explained by the nature of heat conduction in nano-particles and suspensions, the Brownian motion of nano-particles, the arranged structure at the solid/liquid interface, nano-particle clustering, etc.^39–41^. To date there is no consistent theory to predict the anomalous *λ* enhancement of NFs. Whether the differences are due to the previous reasons cited or to any other cause, it is still an open question worthy of further investigation both experimentally and theoretically^42,43^.

As stated earlier, the issue of stability in NFs is one of the causes for the observed contradictions in various published papers on *λ* measurements^5^. Achieving a homogenous dispersion with a long-term stability is essential in order to achieve greater thermal properties in the NFs and better heating performance for long periods of time. On the other hand, the bulk of studies in the literature have concentrated on studying *λ* enhancement in NFs using nano-additives with a much higher *λ* than the base fluids. The present research study is intended to investigate both the thermal conductivity, *λ*, and thermal diffusivity, *a*_*T*_, of very stable fullerene C_60_ based NFs prepared exclusive of any surfactant or dispersing agent. C_60_ has a spherical cage-like fused-ring structure that exhibits a different bond structure of carbon than the previously cited CbNFs and a slightly greater *λ* (0.4 W/m K)^19,44^ than the selected two base liquids, 1,2,3,4-tetrahydronaphthalene (C_10_H_12_) and 1,2-dicholorobenzene (C_6_H_4_Cl_2_). The C_60_ content in the NFs was up to 0.83 vol% in C_10_H_12_ and 1.64 vol% in C_6_H_4_Cl_2_. The measurements were carried out in a wide range of temperatures (254 – 323 K) at atmospheric pressure using the transient multi-current hot wire technique, recognized as the most reliable and precise method to measure *λ* of fluids as it is not adversely affected by convection^45,46^. The *λ* experimental results were compared to the theoretical predictions using different available theoretical models proposed to explain the atypical *λ* enhancement of NFs. The *a*_*T*_ of the two base liquids used in the present study and C_60_ NFs has not yet been systematically studied.

### Thermal conductivity predicted models

One of the curious issues about the *λ* augmentation of NFs is whether the noted changes can be clarified by the existing effective-medium theoretical models. Various useful analytical models have been established to predict the actual *λ* of NFs; whether the nano-additive be spherical, cylindrical, or sheet based^10,43^. The most popular models for NFs like Maxwell, Bruggeman, and Timofeeva models, consider the nano-additive content and its *λ* together with that of the base fluid^37,38,47,48^. The known Brownian motion of micrometer or millimeter nano-additives in suspensions is commonly neglected due to their large size. Nonetheless, its effect was proved to have some impact on *λ* of NFs because of the involved micro-convection of the fluid surrounding the nano-additives. Some thermal conductivity computer models of NFs consider, for instance, the random Brownian motions through some factors influencing its intensity like temperature, particle size, and density^10^.

Various models have taken into consideration the influence of convective heat transfer caused by Brownian motion; such as, Sohrabi’s, Koo and Kleinstreuer's, Bhattacharya’s, and Xue's models^49–52^. Furthermore, in other models it was claimed to take into consideration the effect of a nano-particle’s clustering; such as, Prasher's, Wang's, and Evans' models^53–55^. Further model developments consider the effect of the interfacial nano-layer^51,52,56–59^.

It is apparent in all the previously proposed computer models that the particle volume fraction (*ϕ*), and *λ* of the nano-additive and base fluids should be considered. It is worth noting that the first commonly regarded computer model to predict *λ* of different types of suspensions/solutions is the Maxwell model^37^. This computer model was established for suspensions having low concentrations of homogeneously dispersed, hard spherical particles, and with no interactions between particles as follows^10^:

1 $${\lambda }_{eff}={\lambda }_{f}\frac{{\lambda }_{p}+2{\lambda }_{f}+2\phi \left({\lambda }_{p}-{\lambda }_{f}\right)}{{k}_{p}+2{\lambda }_{f}-\phi ({\lambda }_{p}-{\lambda }_{f})}$$

where *ϕ* is the volume fraction of the dispersed particles in the base liquid and the subscripts *f* and *p* refer to the base liquid and particle, respectively.

Wasp et al.^10,60^ suggested an analogous thermal conductivity model for NFs for a slightly higher concentration of nanoparticles, expressed as:

2 $${\lambda }_{eff}={\lambda }_{f}\frac{{\lambda }_{p}+2{\lambda }_{f}-2\phi \left({\lambda }_{p}-{\lambda }_{f}\right)}{{\lambda }_{p}+2{\lambda }_{f}-\phi \left({\lambda }_{f}-{\lambda }_{p}\right)}$$

Based on homogenous spherical particles, Bruggeman^61^ proposed the following model:

3 $$\varphi \left(\frac{{\lambda }_{p}-{\lambda }_{eff}}{{\lambda }_{p}+2{\lambda }_{eff}}\right)+\left(1-\phi \right)\left(\frac{{\lambda }_{f}-{\lambda }_{eff}}{{\lambda }_{f}+2{\lambda }_{eff}}\right)=0$$

To solve the above equation, Murshed et al.^62^ simplified the Bruggeman model by offering a direct solution given by:

4 $${\lambda }_{eff}=\frac{1}{4}\left[\left(3\phi -1\right){\lambda }_{p}+\left(2-3\phi \right){\lambda }_{f}\right]+\frac{{\lambda }_{f}}{4}\sqrt{\Delta }$$

where $$\Delta ={\left(3\phi -1\right)}^{2}{\left(\frac{{\lambda }_{p}}{{\lambda }_{f}}\right)}^{2}+{\left(2-3\phi \right)}^{2}+2\left(2+9\phi -9{\phi }^{2}\right)\left(\frac{{\lambda }_{p}}{{\lambda }_{f}}\right)$$

The thermal conductivity computer model proposed by Timofeeva et al.^48^ is also built on the effective medium theory and results in:

5 $${\lambda }_{eff}={\lambda }_{f}\left(1+3\phi \right)$$

Xue et al.^51^ proposed a computer model to calculate *λ* of NFs based on carbon nano-tubes (CNT):

6 $${\lambda }_{eff}={\lambda }_{f}\frac{1-\phi +2\phi \frac{{\lambda }_{p}}{{\lambda }_{p}-{\lambda }_{f}}\mathrm{ln}\frac{{\lambda }_{p}+{\lambda }_{f}}{2{\lambda }_{f}}}{1-\phi +2\phi \frac{{\lambda }_{f}}{{\lambda }_{p}-{\lambda }_{f}}\mathrm{ln}\frac{{\lambda }_{p}+{\lambda }_{f}}{2{\lambda }_{f}}}$$

An apparent shortcoming is that the above computer models observably underestimate *λ* of those NFs prepared with nano-particles with a high *λ*. This is owed to the fact that the calculated theoretical *λ* increases with an increase in ${\lambda }_{p}/{\lambda }_{f}$ when the value is below 10 in the Maxwell, Wasp, and Bruggeman models. Conversely, an increase in the rate of the computed *λ* value slowly becomes moderate with an increase in the ${\lambda }_{p}/{\lambda }_{f}$ ; specifically, when the ${\lambda }_{p}/{\lambda }_{f}$ exceeds 20, the calculated *λ* of the NF continues nearly constant with an increase in ${\lambda }_{p}/{\lambda }_{f}$. Other methods, as the Timofeeva model, does not consider *λ* of the nano-particles; which alleviates that particular problem but causes others. Thus, those models do not seem appropriate for NFs created with nanoparticles with a high *λ*.

Xuan et al.^63^ established a computer model that considers the Brownian motion of the nano-additives and clusters together with Maxwell`s model:

7 $${\lambda }_{eff}={\lambda }_{f}\frac{{\lambda }_{p}+2{\lambda }_{f}+2\phi \left({\lambda }_{p}-{\lambda }_{f}\right)}{{\lambda }_{p}+2{\lambda }_{f}-\phi \left({\lambda }_{p}-{\lambda }_{f}\right)}+\frac{1}{2}{\rho }_{p}{c}_{p}\phi \sqrt{\frac{{K}_{B}T}{3\pi {\mu }_{f}{R}_{cl}}}$$

where K_B_ is Boltzmann constant, *R*_*cl*_ is the mean radius of gyration of the cluster, *µ*_*f*_ is the viscosity of the base liquid, *ρ*_*p*_ is the density of the nano-additive, and *c*_*p*_ is its specific heat. Because the second term of Eq. (7) is not dimensionally homogeneous.

(i.e. must be in W/m K), this equation was revised by assigning the unit (m/s^1^^/^^2^) to the constant $(\frac{1}{2\sqrt{3\pi }})$ ^64^.

## Results

**Figure 1:**
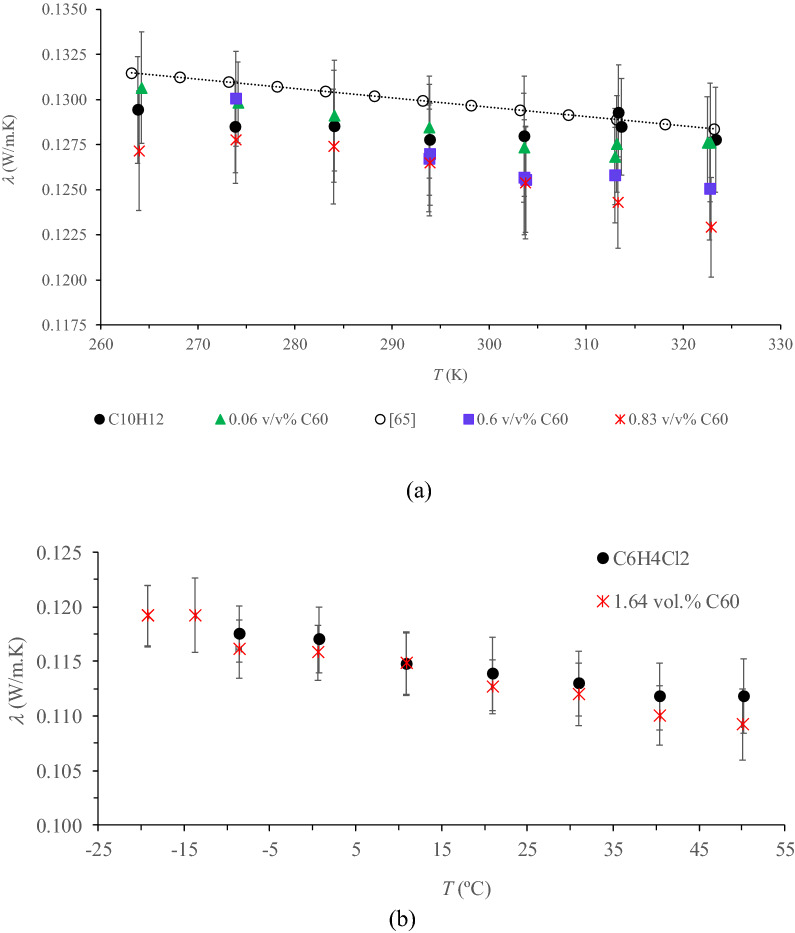
Measured *λ* of C_60_ NFs and base liquids C_10_H_12_ (**a**) and C_6_H_4_Cl_2_ (**b**) as a function of temperature (*T*). Microsoft Excel 2016 was used to generate these figures.

The obtained *λ* of the base fluids 1,2,3,4-tetrahydronaphthalene (C_10_H_12_) and 1,2-dicholorobenzene (ortho-dicholorobenzene, C_6_H_4_Cl_2_) are plotted in Fig. 1 as a function of temperature along with the published literature data for C_10_H_12_^65^. The presented standard deviations are related to 116–411 readings for each temperature. It can be seen that the reference data for C_10_H_12_^65^ is slightly higher than the measured values (< 2%) but within the margin of error confirming the adequate measurements of the hot wire. It is worth noting that the published *λ* values of C_10_H_12_ were given without inclusion of the corresponding associated errors and the followed measurement technique; while for C_6_H_4_Cl_2_ it is unfeasible to make any comparison due to the scarcity available data. For C_6_H_4_Cl_2_, *λ* decreases gradually with the increase of temperature being the slope −1.065 10^–4^ W/m K^2^. However, for C_10_H_12_, no clear decrease of *λ* with temperature was observed, provided that the slope of the reported data was very small −5.205 10^–5^ W/m K^2^. Within the full temperature range studied, *λ* of C_10_H_12_ is higher than that of C_6_H_4_Cl_2_ (9.8–15.7% for 273.9–313.4 K).

**Figure 2:**
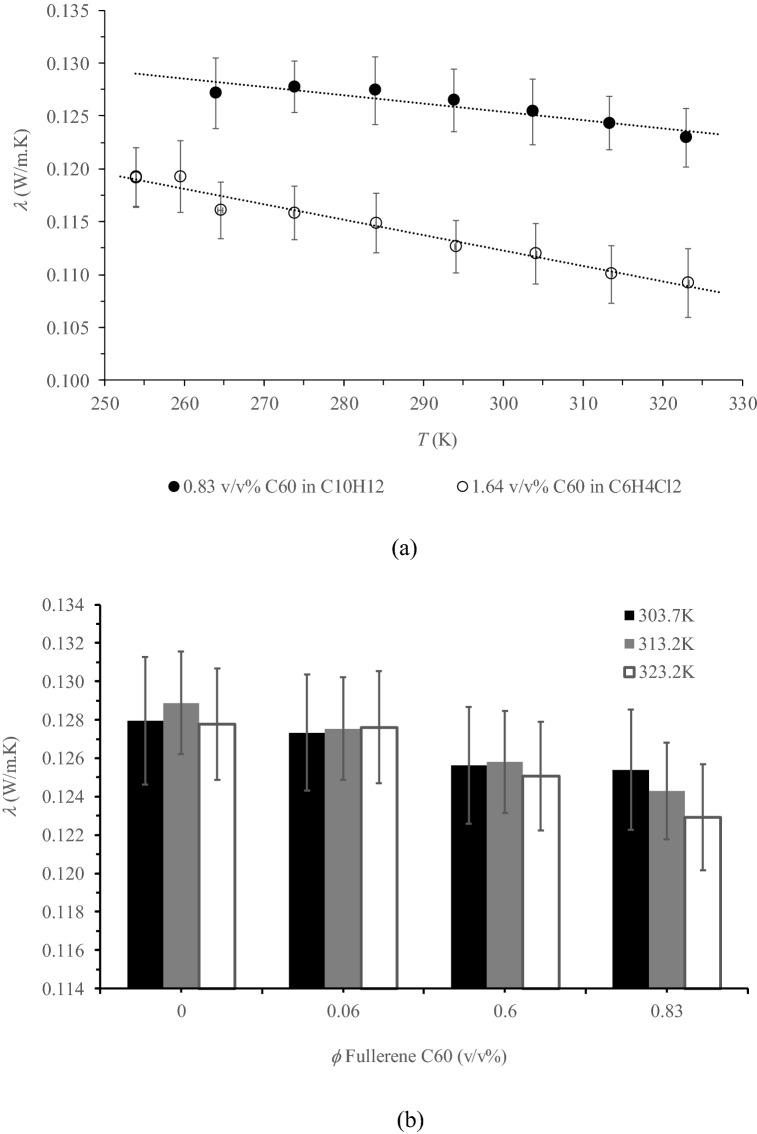
Effect of temperature on *λ* of NFs prepared with 0.83 vol% in C_10_H_12_ and 1.64 vol% in C_6_H_4_Cl_2_ (**a**) and effect of C_60_ concentration (*ϕ*) on *λ* of NFs prepared with C_10_H_12_ for different temperatures (*T*) (**b**). Microsoft Excel 2016 was used to generate these figures.

Similar to the base liquids (solvents), as shown in Fig. 2a, *λ* of the prepared NFs decreased with temperature, indicated by the slope being greater for C_60_ based C_6_H_4_Cl_2_ NF (−1.46 10^–4^ W/m K^2^) than for C_60_ based C_10_H_12_ (−8.45 10^–5^ W/m K^2^). Upon the addition of fullerene C_60_ in C_6_H_4_Cl_2_ (Fig. 2b), a slight reduction of *λ* was observed for the entire temperature range studied (−2.4% reduction for 1.64 vol% C_60_), while for C_10_H_12_ based NFs, as is depicted in Fig. 2b, a slow decrease in *λ* was observed with greater C_60_ concentration but only for temperatures above 303 K (2 , 3.6 and 3.8% reduction for 0.83 vol% C_60_ in C_10_H_12_ at 303.7, 313.2 and 323.2 K, respectively). This contradicts the previously reported *λ* enhancement of various types of carbon-based nano-additives (graphene, carbon nanotubes, nano-diamond particles, etc.)^2,3,5,9–27,29,35,41^; although *λ* of C_60_ (0.4 W/m K) is approximately 3.1% and 3.5% higher than that of the based liquids C_10_H_12_ and C_6_H_4_Cl_2_, respectively. In fact, *λ* of various types of carbon based nano-additives is extremely greater than that of C_60_ (e.g. 5300 W/m K for graphene^8^, 6000 W/m K for single walled carbon nano-tubes^7^, 3986 W/m K for double walled carbon nano-tubes^7^, and 3000 W/m K for multi-walled carbon nano-tubes^5,7,9^). In comparison to the base liquid, water, Hwang et al.^28^ also observed a lower *λ* value for water based C_60_ NFs, and *λ* decreased with a greater C_60_ volume fraction. For the same base liquid, water, MWCNT NFs exhibited greater *λ* values than that of water (e.g. For MWCNT NF, *λ* increased by approximately 7.0% at a volume fraction of 1.0%; whereas for C_60_ it decreased by approximately 3.0% for a volume fraction of 1.5%). In this case, *λ* of a C_60_ water based NF is less than that of water. For oil-based liquids, *λ* was improved for both C_60_ and MWCNT NFs (e.g. *λ* increased by 6.0% for 5 vol% of C_60_ and increased by 8.7% for 0.5 vol% of MWCNT). It was found that *λ* of MWCNT NF was considerably better than that of a C_60_ NF because *λ* of MWCNT is superior than that of C_60_^28^. For the same nano-additive loading, it is generally thought that the nano-additive having a higher *λ* induces a greater *λ* enhancement of the NFs. However, this conclusion is uncertain for specific types of NFs, since *λ* of the NFs is concurrently influenced by numerous other things such as aggregation, Brownian motion, interfacial nano-layer, surface charge state, and the thermal resistance of the nanoparticles^10^. More theoretical studies should be conducted in order to accurately reveal the governing mechanism(s) and justify the reduction in *λ* observed in our study.

**Figure 3:**
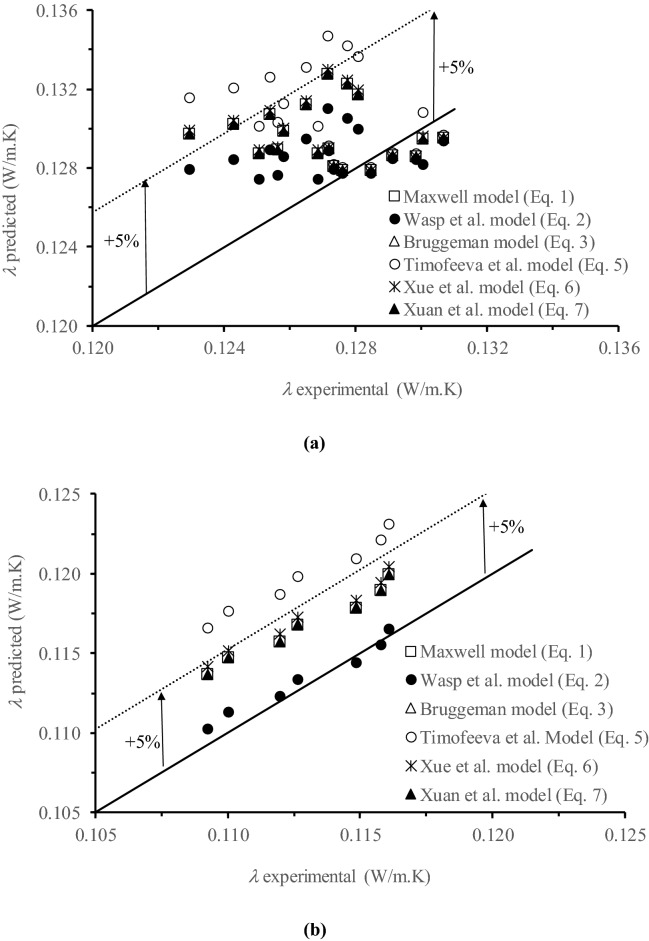
Predicted *λ* of C_60_ NFs based C_10_H_12_ (**a**) and in C_6_H_4_Cl_2_ (**b**) vs. the obtained experimental *λ* values. Microsoft Excel 2016 was used to generate these figures.

As stated in the previous section thermal conductivity predicted models, the *λ* of the prepared NFs can be predicted using the mentioned theoretical models [Eqs. (1–7)]. The calculated *λ* values were plotted against the experimental ones in Fig. 3. In general, good agreements were observed for all models (Maxwell model [Eq. (1)], Wasp et al. model [Eq. (2), Bruggeman model [Eq. (3)], Timofeeva et al. model [Eq. (5)], Xue et al. model [Eq. (6)], Xuan et al. model [Eq. (7)] within 5% confidence except for the Timofeeva et al. model [Eq. (5)], which showed more than 5% deviation from the experimental values (i.e. maximum registered deviations were up to 7%). For small concentrations in volume fraction of C_60_ in C_10_H_12_ (< 0.6 vol.%), the calculated *λ* values from the Timofeeva et al. model agree well with the experimental one. However, for higher concentrations, this model overestimated *λ*. These conclusions are attributed to the fact that, in contrast to the other theoretical models, the Timofeeva et al. model does not consider *λ* of the nano-additive C_60_. Nearly the same deviations from the experimental data were obtained for the Maxwell, Bruggeman, and Xuan et al. models. The latter model (Xue) is based on the Maxwell model and the influence of Brownian motion of C_60_ was found to be negligible (4.2 10^–4^—6.7 10^–4^% for 1.64 vol% of C_60_ based C_6_H_4_Cl_2_ NF and 2.6 10^–5^ – 1.2 10^–4^% for 0.06 vol% of C_60_ based C_10_H_12_ NF, 2.6 10^–4^—1.2 10^–3^% for 0.6 vol% of C_60_ based C_10_H_12_ NF and 3.4 10^–4^ – 1.6 10^–3^% for 0.83 vol% of C_60_ based C_10_H_12_ NF). It should be mentioned that the contribution of Brownian motion of C_60_ increased with an increase in C_60_ content within the based fluid and with temperature, as can be expected from Eq. (7); although the obtained values were very low. The observed negligible influence of Brownian motion of C_60_ in both BLs, justifies the similarity of the registered temperature reliance of the *λ* of the NFs to the BLs. In fact, Brownian motion increases *λ* of NFs whereas the influence of aggregation is adverse for Brownian motion^10^. Some researchers reported a negative effect on *λ* when increasing the temperature. For SiO_2_ based water NFs, Masuda et al.^66^ observed a *λ* enhancement of approximately 10–11% at 31.85 °C, 9–10% at 46.85 °C, and 5–7% at 66.85 °C when the volume loading was increased from 1.1 to 2.3%. This indicates the different influences of temperature on *λ* of NFs through particle Brownian motion, dispersion stability, and particles clustering. In this study, because of the negligible Brownian motion of C_60_ and stability of the prepared NFs, the base liquids dictated the temperature dependency of the NFs (i.e. similar trends were observed for the base liquid and the corresponding NFs).

Taking into consideration the deviations of all data for both based liquids, among the considered model, Wasp et al. model^60^ is the best one (i.e. the obtained deviations from the measured values were −0.4 to 1.1% for 1.64 vol% of C_60_ in C_6_H_4_Cl_2_ NF; −0.6 −1.1% for 0.06 vol% of C_60_ in C_10_H_12_ NF; −1.5 to 2.2% for 0.6 vol% of C_60_ in C_10_H_12_ NF and 1.5—4.1% for 0.83 vol% of C_60_ in C_10_H_12_ NF).

**Figure 4:**
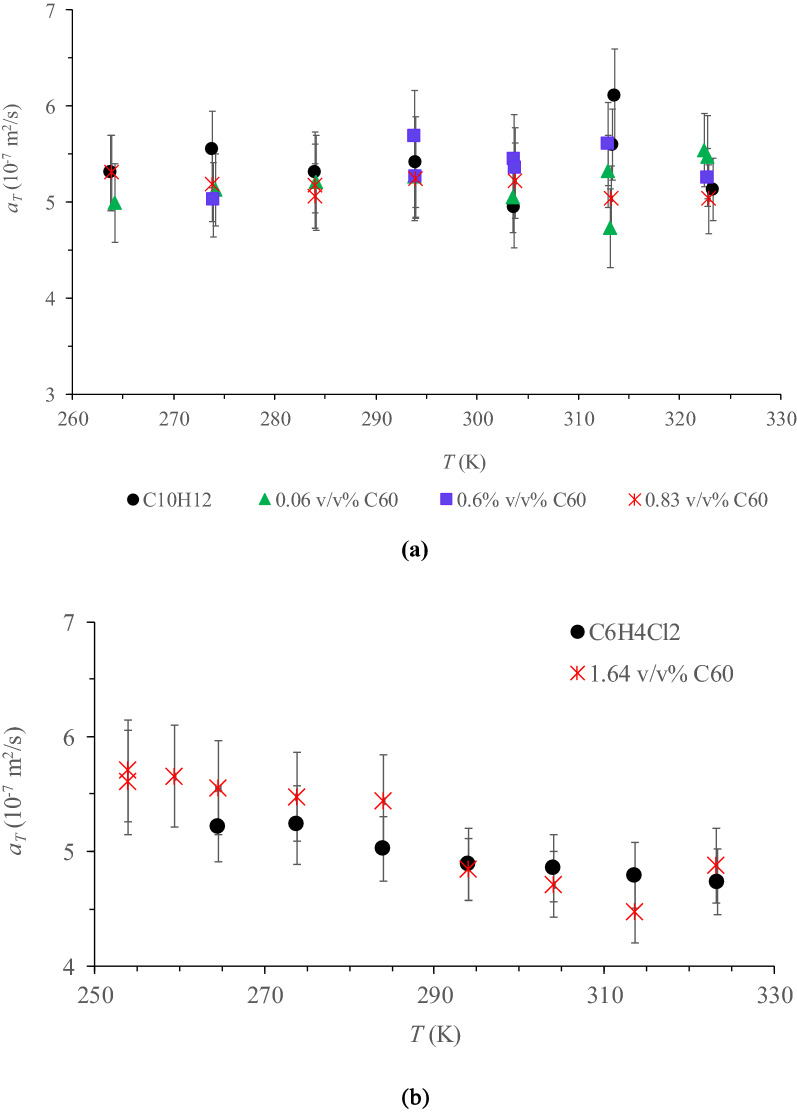
Measured *a*_*T*_ of C_60_ NFs together with the corresponding base liquids C_10_H_12_ (**a**) and in C_6_H_4_Cl_2_ (**b**) as a function of temperature (*T*). Microsoft Excel 2016 was used to generate these figures.

Apropos that there is not any data for *a*_*T*_ that has been reported for the base liquids used in this study, this was determined by the hot wire technique as explained in Measurements section, for both C_10_H_12_ and C_6_H_4_Cl_2_ together with the above mentioned C_60_ NFs. The results are plotted in Fig. 4 as a function of temperature, along with their associated inaccuracies.

For C_10_H_12_, taking into consideration the standard errors, no clear variation of *a*_*T*_ was detected with neither a change in temperature nor with the addition of C_60_. As indicated in our previous study^67^, the uncertainty associated to *a*_*T*_ (up to 9.5%) acquired by the transient hot-wire method is greater than that of *λ* (up to 3%). However, a minor decrease with temperature was observed for C_6_H_4_Cl_2_ (i.e. 9.3% when the temperature was increased from 264.6 to 323.3 K). Similarly, as with C_10_H_12_, no variations were observed between *a*_*T*_ values of C_6_H_4_Cl_2_ and the C_60_ related NFs for the entire temperature range studied. This result may be attributed partly to the small variation of the *λ* upon the addition of C_60_ and with temperature $({a}_{T}=\frac{\lambda }{{\rho c}_{P}})$.

## Discussion

For the first time, very stable fullerene (C_60_)-based NFs were prepared without any surfactant or dispersing agent using two base liquids C_10_H_12_ and C_6_H_4_Cl_2_; and both *λ* and *a*_*T*_ were measured in liquid phase by the transient multi-current hot wire technique at atmospheric pressure in a wide range of temperature (254 – 323 K).

The obtained *λ* of C_10_H_12_ in this study is in very good accord with the values found from the only reported ones 27 years ago in^65^. However, for C_6_H_4_Cl_2_, it is impossible to produce any such comparison due to the lack of published data.

Similar to the base liquids, *λ* of both types of C_60_ NFs decreased slightly with an increase in temperature. However, in contrast to what it was expected (compared to the base liquids), *λ* of the C_60_ NFs was reduced upon the addition of C_60_ and decreased with an increase in its concentration. The obtained experimental *λ* values were compared to the values predicted using different theoretical thermal conductivity models, originally proposed to clarify the abnormal improvement of *λ* of NFs. In general, good accord was found, within 5% confidence, with the experimental data for the Maxwell [Eq. (1)], Wasp et al. [Eq. (2)], Bruggeman [Eq. (3)], Xue et al. [Eq. (6)], and Xuan et al. models [Eq. (7)]. More than 5% deviation, and up to 7%, was observed for the Timofeeva et al. model [Eq. (5)], mainly because of the overestimation of *λ* at high C_60_ concentrations in the NFs; ascribed to the fact that this model does not consider *λ* of the nano-particles. In general, the Brownian motion of C_60_ was found to be negligible in the studied NFs.

It was not possible to find any literature data for *a*_*T*_ of C_10_H_12_ and C_6_H_4_Cl_2_ to be used in this study. For the tested base liquids, no clear variation of *a*_*T*_ was detected between the C_60_ NF and the corresponding base liquid for the entire temperature range studied. This was partly due to the small variation of *λ* upon the addition of C_60_. For C_10_H_12_ no clear tendency was observed between *a*_*T*_ and the temperature. However, a slight decrease (9.3%) was observed for C_6_H_4_Cl_2_ when the temperature was increased from 264.6 to 323.3 K.

## Conclusions

There has been a significant amount of research on *λ* enhancement of different types of NFs, but the results differ even for the same NF. More importantly, the large enhancement in *λ* of NFs prepared with a small amount of nano-additive is regarded as anomalous and controversial. We measured for the first time both *λ* and *a*_*T*_ of two types of stable fullerene-based NFs together with their base liquids, 1,2,3,4-tetrahydronaphthalene and 1,2-dicholorobenzene, by the transient multi-current hot wire technique at atmospheric pressure and different temperatures in the range 254 – 323 K.

We found that the obtained *λ* of 1,2,3,4-tetrahydronaphthalene is in very good agreement with the values reported in the literature more than 25 years ago confirming the adequacy of the developed hot-wire measurements. However, we did not find any literature data for *λ* of 1,2-dicholorobenzene. Similarly to the base liquids, we found that *λ* of both types of C_60_ NFs decreased slightly with the increase of the temperature. However, in contrast to what we expected, compared to the base liquids, *λ* of the NFs was reduced upon the addition of fullerene. We compared the obtained results with the predicted ones using different thermal conductivity theoretical models and good agreement was observed between them (up to 7% deviation). More theoretical studies should be conducted in order to accurately reveal the governing mechanism(s) and justify the reduction in *λ* observed in this research study, rather than the anomalous *λ* enhancement of NFs.

We didn’t find in the literature *a*_*T*_ data of fullerene-based NFs or the base liquids 1,2,3,4-tetrahydronaphthalene and 1,2-dicholorobenzene. We didn’t detect much variation in *a*_*T*_ between the C_60_ NFs and the corresponding base liquids tested at different temperatures due partly to the small variation of *λ* with the addition of C_60_.

## Methods

### Materials and preparation of nanofluids

The based liquids used to prepare the NFs, 1,2,3,4-tetrahydronaphthalene (C_10_H_12_) and 1,2-dicholorobenzene (ortho-dicholorobenzene, C_6_H_4_Cl_2_) having minimum purities of 99% were supplied by Sigma-Aldrich. The solvents were used without further purification or removal of any dissolved water or air. Fullerene C_60_ with approximately 0.7 nm in diameter and 99.5% purity was purchased from Sigma-Aldrich. To calibrate the *λ* measurements of the wire, dimethyl phthalate (C_10_H_10_O_4_, 99% purity, Sigma- Aldrich) with a known *λ* value was used following the ASTM D2717 standard test^68^.

The method commonly followed for NF preparation is known as the two-step method. With this method, the preparation procedures of nano-additive and NF are implemented independently. In this study, dry C_60_ was suspended into the base liquid, first stirred for 30 min at 150 rpm (Ika RCT basic) and then subjected to ultra-sonication for multiple 30-min intervals in order to remove any possible aggregations or colliding nanoparticles due to their high activity and interaction force. Then the dispersion was left overnight to assure that there was no precipitation of the nanoparticles. When the C_60_ nanoparticles were introduced to the clear base fluids it turned them a very dark purple/violet. The highest possible C_60_ loading in C_6_H_4_Cl_2_ was 2 wt% (1.64 v/v%) while in C_10_H_12_ it was found to be lower 1.35 wt% (0.83 v/v%). It is worth noting that no fullerene settlements were observed during the whole experimental time after NF preparation and even after all testing periods, so the fraction of contained C_60_ remained unchanged. No clear variation of *λ* with time was detected indicating very good dispersion stability of the prepared samples.

### Measurements

The transient hot-wire method is the most common method to quantify *λ* of different types of materials of both liquid and solid phases^45,46,67^. In this study, both *λ* and *a*_*T*_ were measured using the transient hot wire experimental apparatus detailed in our previous studies^67,69,70^. The followed method is similar to the ones explained for the measurement of *λ* of several fluids^67,69,70^. Briefly, a platinum wire of 50 ± 0.001 μm diameter and 5.92 ± 0.07 cm length was used. Each end of the platinum wire was first soldered to tabs on a properly prepared, chemically resistant, flat frame, cut from a raw circuit board (substrate). Two leads (Teflon jacketed and isolated) were then soldered to each tab; the soldered tabs were subsequently covered by a thermal resistant epoxy. Before calibration a Teflon-based industrial coating was applied to the platinum wire having a thickness layer of less than 1 μm in order to prevent any electrical connection among the platinum wire and the sample.

The liquid sample is placed inside a cylindrical double-walled glass cell that has an internal diameter of 2.2 cm, an external diameter of 4.1 cm and a height of 13 cm. The temperature of the glass cell was controlled within ± 0.05 K by means of a working fluid, which flowed from a thermostatic bath (Lauda ECO RE630) through the jacket of the glass cell. The temperature of the samples were measured by a platinum resistance thermometer (F250 MKII Precision Thermometer, accuracy <  ± 0.005ºC), which together with the hot wire, were inserted inside the glass cell containing the sample. The whole measurement cell was kept inside a climatic chamber (Mytron) set at the same temperature of the test temperature maintaining the humidity to around 40%.

To conduct the electrical measurements, we used a Keithley 2400 source-meter that allows operation as a current source and voltage meter simultaneously. The acquisition equipment was then connected to a desktop computer and a software code was developed to run the experimental test, register the measurement points, conduct a data fitting, and finally calculate *a*_*T*_ and *λ*. The platinum thermometer was also coupled to the computer. In this study, the applied electrical currents (200, 250 and 300 mA) were first applied through two of the leads connected to the wire ends, while the voltage was measured concurrently by the other two leads.

**Figure 5:**
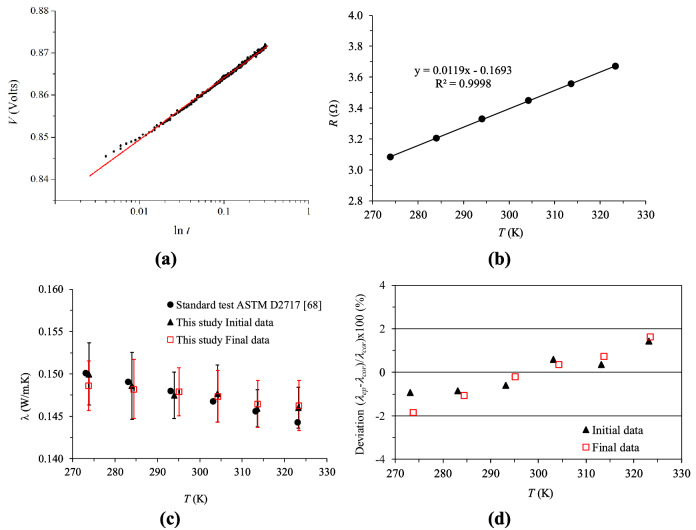
Typical discrete heating curve showing the voltage drop in the platinum wire, *V*, as a function of time for the C_60_ NF prepared by dispersing 0.6 v/v% in C_10_H_12_ at 293.15 K and 250 mA (The straight line represent the fitting to Eq. (8)) (**a**), variation of the wire resistance, *R,* with temperature, *T* (**b**), measured *λ* of C_10_H_10_O_4_ as a function of *T* following the ASTM D2717 standard test^68^ to calibrate the wire before (Initial data) and after (Final data) testing base liquids and NFs (**c**) and deviation from the tabulated values of the measured *λ* (**d**). Microsoft Excel 2016 was used to generate these figures.

To ensure the satisfactory measurements of the wire, first experimental runs using C_10_H_10_O_4_ at different temperatures were performed and the obtained *λ* values were compared to the reported ones following the ASTM D2717 standard test^68^ (See Fig. 5c,d). For each temperature of the sample, when steady-state has been established, the software was run under the established electrical currents, which were administered to the wire every 4 min. The recorded data consisted of time (*t*), the electric potential (*V*), the temperature (*T*), and the resistance of the wire (*R*_*0*_) at the beginning of the heating step. With each measurement, approximately 350 values of voltage (*V*) as a function of time were recorded and the measurements were performed for at least 6 and as many as 16 h depending on the set temperature. A typical heating run lasts for approximately 0.34–1.6 s (depending on the record rate). Figure 5a shows as an example the variation with time of the electric potential between the wire ends.

Due to Joule heating, when an electrical current (*I*) is applied across the platinum wire, the temperature (*T*), resistance (*R*), and voltage difference (Δ*V*) of the wire increases depending on *λ* and *a*_*T*_ of the sample surrounding the wire. For an infinite cylindrical straight wire, the change with time (*t*) of the voltage difference (Δ*V*) between two points separated by a distance (*L*) can be approximated for large time, $t > > \frac{{r_{0}^{2} \rho^{{}} c_{p} }}{4\lambda }$, by the following equation as reported elsewhere^40,71,72^.

8 $$ \Delta V(t) = V(t) - V_{0} = \alpha \frac{{I^{3} R_{0}^{2} }}{4\pi \lambda L}\left[ {\ln \left( {\frac{{4\lambda^{{}} t}}{{r_{0}^{2} \rho^{{}} c_{p} }}} \right) - \gamma } \right] $$

where *α* is the temperature resistance coefficient of the Platinum wire, *R*_0_ is the electrical resistance of the wire at the beginning of the heating (*t* = 0), *γ* is Euler’s constant (*γ* = 0*.*5772), and *L* is the length of the wire. Equation (8) can be rewritten as:

9 $$ V(t) = m\frac{{I^{3} R_{0} }}{4\pi \lambda L}\left[ {\ln \left( {\frac{t}{\beta }} \right) - \gamma } \right] + V_{0} $$

where *m* is the slope of the {*R ,T*} curve at the initial temperature of the heating run (see Fig. 5b) and the parameter, *β*, (units of time) depends on *a*_*T*_ of the sample and the wire radius (*r*_*o*_) as follows:

10 $$ \beta = \frac{{r_{0}^{2} }}{{4a_{T} }} $$

The resistance (*R*) is recorded for each measurement, temperature, sample, and electrical current. A mean value is calculated and the variation in resistance (*R*) with temperature (*T*) is plotted in Fig. 5b. The calculated slope (*m*) value is 0.0119 Ω/K.

*λ* of the sample is calculated from Eq. (9) as follows: the solid line in Fig. 5a depicts the fitting of the experimental points {*V*_*i*_* ,* ln(*t*_*i*_)} to Eq. (9) when the system reaches steady-state (i.e. *t* >  > 412 ms; the first 150 points have not been utilized in determining the fitting). It must be pointed out that deviations in this study were not observed between the measured data and the straight lines in the long time asymptotic regime indicating that the measurements are free of natural convection. From the obtained intercept (*B*) and slope (*S*) of the straight lines, *λ* and *a*_*T*,_ were determined using the following expressions:

11 $$ \lambda = \frac{{m^{{}} I^{3} R_{0} }}{{4\pi L^{{}} S}} = A\frac{{I^{3} R_{0} }}{S} $$

12 $$ a_{T} = \frac{{r_{0}^{2} }}{{4_{{}} }}\exp \left( {\frac{{B - V_{0} }}{S} + \gamma } \right) $$

where *A* is a constant of the wire that is determined based on its effective length. For the hot wire used in this study, *A* was found to be 0.015848 Ω/K.m. This was determined from the measured and reported *λ* values of dimethyl phthalate at different temperatures. The effective length of the wire (*L*_*eff*_) was calculated from the equation (*L*_*eff*_ = *m*/(4π*A*)). The calculated value of 5.975 cm is very close to the measured length of the wire 5.92 ± 0.07 cm (1.0% deviation). This confirms that the measured *a*_*T*_ and *λ* data in this investigation are absolute values. It is worth noting that the behavior of the electrical resistance of the platinum wire with temperature permits verification to the stability of the wire during the measurement run. Additionally, variances were not observed between the tests performed with the samples at the beginning, at the end, nor when switching between tested NFs.

## Availability of Data

All data generated or analysed during this study are included in this published article [and its supplementary information file].

## Supplementary Information

Supplementary Information.
