# Supplementary material for: Thermal conductivity and thermal diffusivity of fullerene-based nanofluids
Source: Sci Rep. 2022 Jun 10;12:9603. doi: 10.1038/s41598-022-14204-y (PMC9187655; doi:10.1038/s41598-022-14204-y)
Supplement: Supplementary file 1 — Supplementary Information. [file 41598_2022_14204_MOESM1_ESM.docx]

**Supplementary Information**

# Thermal conductivity and thermal diffusivity of fullerene-based nanofluids

### Brian Reding, Mohamed Khayet^*^

Department of Structure of Matter, Thermal Physics and Electronics, Faculty of Physics, University Complutense of Madrid, Avda. Complutense s/n, 28040, Madrid (Spain).

^*^Corresponding author (M. Khayet) [khayetm@fis.ucm.es](mailto:khayetm@fis.ucm.es) ; Tel. 91-394-5185

**Table S1.** Thermal conductivity, *λ*, of the base liquid 1,2,3,4-tetrahydronaphthalene (C_10_H_12_) **(a)**, C_60_ NFs based C_10_H_12_ (0.06 v/v% C60; 0.6 v/v% C60; and 0.83 v/v% C60) **(b)**, base liquid 1,2-dicholorobenzene (C_6_H_4_Cl_2_) and C_60_ NF based C_6_H_4_Cl_2_ (1.64 v/v% C60) **(c)** for different temperatures.

**(a)**

| **C_10_H_12_** | |
| --- | --- |
| *T*  (K) | *λ*  (W/m.K) |
| 263.79 | 0.1294 ± 0.003 |
| 273.86 | 0.1285 ± 0.003 |
| 284.02 | 0.1285 ± 0.003 |
| 293.87 | 0.1278 ± 0.003 |
| 303.60 | 0.1280 ± 0.003 |
| 313.33 | 0.1293 ± 0.003 |
| 313.63 | 0.1285 ± 0.003 |
| 323.31 | 0.1278 ± 0.003 |

**(b)**

| **0.06 v/v% C60** | | **0.6 v/v% C60** | | **0.83 v/v% C60** | |
| --- | --- | --- | --- | --- | --- |
| *T*  (K) | *λ*  (W/m.K) | *T*  (K) | *λ*  (W/m.K) | *T*  (K) | *λ*  (W/m.K) |
| 264.20 | 0.131 ± 0.003 | 273.92 | 0.130 ± 0.003 | 263.93 | 0.127 ± 0.003 |
| 274.13 | 0.130 ± 0.002 | 293.82 | 0.127 ± 0.003 | 273.88 | 0.128 ± 0.002 |
| 284.05 | 0.129 ± 0.003 | 293.89 | 0.127 ± 0.003 | 283.97 | 0.127 ± 0.003 |
| 293.85 | 0.128 ± 0.003 | 303.62 | 0.126 ± 0.003 | 293.87 | 0.127 ± 0.003 |
| 303.58 | 0.127 ± 0.003 | 303.76 | 0.126 ± 0.003 | 303.75 | 0.125 ± 0.003 |
| 312.97 | 0.127 ± 0.003 | 312.98 | 0.126 ± 0.003 | 313.29 | 0.124 ± 0.003 |
| 313.16 | 0.128 ± 0.003 | 322.73 | 0.125 ± 0.003 | 322.88 | 0.123 ± 0.003 |
| 322.48 | 0.128 ± 0.003 |  |  |  |  |
| 322.80 | 0.128 ± 0.003 |  |  |  |  |

**(c)**

| **C_6_H_4_Cl_2_** | | **1.64 v/v% C60** | |
| --- | --- | --- | --- |
| *T*  (K) | *λ*  (W/m.K) | *T*  (K) | *λ*  (W/m.K) |
| 264.54 | 0.118 ± 0.003 | 253.97 | 0.119 ± 0.003 |
| 273.81 | 0.117 ± 0.003 | 253.96 | 0.119 ± 0.003 |
| 284.01 | 0.115 ± 0.003 | 259.49 | 0.119 ± 0.003 |
| 294.02 | 0.114 ± 0.003 | 264.54 | 0.116 ± 0.003 |
| 304.11 | 0.113 ± 0.003 | 273.77 | 0.116 ± 0.003 |
| 313.56 | 0.112 ± 0.003 | 284.07 | 0.115 ± 0.003 |
| 323.31 | 0.112 ± 0.003 | 294.05 | 0.113 ± 0.002 |
|  |  | 304.13 | 0.112 ± 0.003 |
|  |  | 313.61 | 0.110 ± 0.003 |
|  |  | 323.26 | 0.109 ± 0.003 |

**Table S2.** Thermal diffusivity, *a_T_*, of the base liquid 1,2,3,4-tetrahydronaphthalene (C_10_H_12_) **(a)**, C_60_ NFs based C_10_H_12_ (0.06 v/v% C60; 0.6 v/v% C60; and 0.83 v/v% C60) **(b)**, base liquid 1,2-dicholorobenzene (C_6_H_4_Cl_2_) and C_60_ NF based C_6_H_4_Cl_2_ (1.64 v/v% C60) **(c)** for different temperatures.

**(a)**

| **C_10_H_12_** | |
| --- | --- |
| *T*  (K) | *a_T_*  (10^-7^ m^2^/s) |
| 263.79 | 5.30 ± 0.39 |
| 273.86 | 5.55 ± 0.40 |
| 284.02 | 5.31 ± 0.42 |
| 293.87 | 5.42 ± 0.48 |
| 303.60 | 4.94 ± 0.41 |
| 313.33 | 5.60 ± 0.37 |
| 313.63 | 6.10 ± 0.49 |
| 323.31 | 5.13 ± 0.33 |

**(b)**

| **0.06 v/v% C60** | | **0.6 v/v% C60** | | **0.83 v/v% C60** | |
| --- | --- | --- | --- | --- | --- |
| *T*  (K) | *a_T_*  (10^-7^ m^2^/s) | *T*  (K) | *a_T_*  (10^-7^ m^2^/s) | *T*  (K) | *a_T_*  (10^-7^ m^2^/s) |
| 264.20 | 4.99 ± 0.41 | 273.92 | 5.02 ± 0.39 | 263.93 | 5.30 ± 0.39 |
| 274.13 | 5.12 ± 0.38 | 293.82 | 5.69 ± 0.47 | 273.88 | 5.19 ± 0.39 |
| 284.05 | 5.20 ± 0.50 | 293.89 | 5.26 ± 0.43 | 283.97 | 5.17 ± 0.44 |
| 293.85 | 5.26 ± 0.45 | 303.62 | 5.44 ± 0.47 | 293.87 | 5.24 ± 0.40 |
| 303.58 | 5.05 ± 0.38 | 303.76 | 5.35 ± 0.42 | 303.75 | 5.22 ± 0.39 |
| 312.97 | 5.32 ± 0.38 | 312.98 | 5.60 ± 0.44 | 313.29 | 5.04 ± 0.34 |
| 313.16 | 4.73 ± 0.41 | 322.73 | 5.25 ± 0.30 | 322.88 | 5.03 ± 0.36 |
| 322.48 | 5.54 ± 0.38 |  |  |  |  |
| 322.80 | 5.47 ± 0.43 |  |  |  |  |

**(c)**

| **C_6_H_4_Cl_2_** | | **1.64 v/v% C60** | |
| --- | --- | --- | --- |
| *T*  (K) | *a_T_*  (10^-7^ m^2^/s) | *T*  (K) | *a_T_*  (10^-7^ m^2^/s) |
| 264.54 | 5.22 ± 0.31 | 253.97 | 5.70 ± 0.44 |
| 273.81 | 5.23 ± 0.34 | 253.96 | 5.60 ± 0.46 |
| 284.01 | 5.02 ± 0.28 | 259.49 | 5.66 ± 0.44 |
| 294.02 | 4.88 ± 0.31 | 264.54 | 5.55 ± 0.41 |
| 304.11 | 4.85 ± 0.29 | 273.77 | 5.48 ± 0.38 |
| 313.56 | 4.79 ± 0.29 | 284.07 | 5.44 ± 0.39 |
| 323.31 | 4.73 ± 0.29 | 294.05 | 4.85 ± 0.27 |
|  |  | 304.13 | 4.71 ± 0.29 |
|  |  | 313.61 | 4.48 ± 0.27 |
|  |  | 323.26 | 4.88 ± 0.32 |
